# The Peculiar H-Bonding Network of 4-Methylcatechol: A Coupled Diffraction and In Silico Study

**DOI:** 10.3390/molecules29102173

**Published:** 2024-05-07

**Authors:** Mattia Lopresti, Luca Palin, Giovanni Calegari, Marco Milanesio

**Affiliations:** 1 Dipartimento di Scienze e Innovazione Tecnologica, Università del Piemonte Orientale, Viale T. Michel 11, 15121 Alessandria, Italy; mattia.lopresti@uniupo.it (M.L.); luca.palin@uniupo.it (L.P.); 2 Nova Res s.r.l., Via D. Bello 3, 28100 Novara, Italy; 3 Enartis s.r.l., Via S. Cassiano 99, 28069 Trecate, Italy

**Keywords:** polyphenol, antioxidant, crystal structure, X-ray powder diffraction, Hirshfeld surface, hydrogen bond, first principle calculation, octagonal H-bond nests

## Abstract

The crystal structure of 4-methylcatechol (4MEC) has, to date, never been solved, despite its very simple chemical formula C_7_O_2_H_8_ and the many possible applications envisaged for this molecule. In this work, this gap is filled and the structure of 4MEC is obtained by combining X-ray powder diffraction and first principle calculations to carefully locate hydrogen atoms. Two molecules are present in the asymmetric unit. Hirshfeld analysis confirmed the reliability of the solved structure, since the two molecules show rather different environments and H-bond interactions of different directionality and strength. The packing is characterised by a peculiar hydrogen bond network with hydroxyl nests formed by two adjacent octagonal frameworks. It is noteworthy that the observed short contacts suggest strong inter-molecular interactions, further confirmed by strong inter-crystalline aggregation observed by microscopic images, indicating the growth, in many crystallization attempts, of single aggregates taller than half a centimetre and, often, with spherical shapes. These peculiarities are induced by the presence of methyl group in 4MEC, since the parent compound catechol, despite its chemical similarity, shows a standard layered packing alternating hydrophobic and polar layers. Finally, the complexity and peculiarity of the packing and crystal growth features explain why a single crystal could not be obtained for a standard structural analysis.

## 1. Introduction

In the world of chemistry, some compounds are like Swiss army knives: incredibly versatile and useful in a variety of ways. One such compound is 4-methylcatechol (4MEC), which plays a pivotal role in numerous industrial and research applications, including as an important ingredient in fragrance and flavoring industries, a crucial intermediate in syntheses, a bioactive component, an excellent model molecule for electrochemical process studies, and a polymerization inhibitor. In addition to playing a central role in many fields of application, it appears to have different biological functions, including positive effects on neuronal tissue, anticoagulants [1], and vasorelaxants [2]. A natural source of 4MEC, can be found in castoreum [3], the exudate from the castor sacs of mature beavers (*Castor canadensis*), which serves multiple purposes in both natural and industrial settings. Beavers utilize castoreum, along with urine, for scent-marking their territory [4]. Extracted from dried and alcohol-tinctured beaver castoreum, it finds application in perfumery, imparting a note reminiscent of leather [5]. Castoreum also serves as a food additive, as it is recognized as safe by regulatory bodies like the Food and Drug Administration in the United States [6]. Its usage in the flavouring industry remains modest compared to other additives, with applications ranging from flavoring schnapps in Sweden [7,8] to potential contributions to the flavor and aroma of cigarettes [9]. Due to its well-established oxidation mechanisms, 4MEC serves as a model molecule in studying the oxidation of systems containing analogous polyphenols, such as catechin in wine [10,11,12]. Its use provides a cost-effective means to investigate the behavior of catechins and related flavonoids, contributing to a deeper understanding of oxidation processes in various contexts. Other applications as model molecule are referred to in studies involving enzymatic browning, which poses challenges in the fruit and vegetable industries, since browning impacts both sensory attributes and nutritional quality [13]. Such browning is caused by polyphenol oxidase (PPO), which catalyzes the polymerization of o-quinones to produce black, brown or red pigments, resulting in fruit browning. 4MEC, alongside other ortho-diols like catechol and chlorogenic acid, serves as a common substrate in studying the activities of PPO enzymes [14,15,16,17,18,19,20], offering insights into enzymatic browning mechanisms. In the scientific literature, the propensity of 4MEC to form adducts with proteins, such as β-Lactoglobulin [21] and also cysteine thiolic residues in meat proteins [22,23,24,25], is reported, highlighting its interaction with molecules of biological importance. Moreover, research indicates its potential in promoting nerve regeneration [26], as evidenced in rodent models of neuropathy and nerve injury [27]. Additionally, its effects on chronic pain and depression have been investigated in studies on mice and rats [28]. In the medical and pharmaceutical realms, the topical application of 4MEC in hydrogel form demonstrates promise in promoting epidermal regeneration [29] and exhibits anti-platelet properties [1,30]. Beyond its biological applications, 4MEC has been evidenced as covering a important role in electrochemical reactions [31], and serving as a model molecule for assessing the performance of electrodes for voltammetry [32,33,34]. Despite these multiple applications and this in-depth knowledge of 4MEC from many points of view, no information is available on its solid-state characteristics, as its crystal structure has never been solved before, despite its very simple chemical formula. This is due to the peculiar difficulty of growing crystals of sufficient size and quality for single crystal X-ray diffraction resolution. In this article, to fill this gap, the structure of 4MEC is resolved by combining X-ray powder diffraction and first principle calculations to study the peculiar hydrogen bonding system. Hirshfeld surface analysis and energy framework calculation were carried out to complete the in silico analysis of the structure. Understanding the interactions and the solid-state molecular features of such a widely used molecule might suggest the behavior of this molecule in solution and also in vivo [35], helping, for instance, molecular docking studies involving 4MEC.

## 2. Results and Discussion

### 2.1. Recrystallization Attempts and Morphology

**Figure 1:**
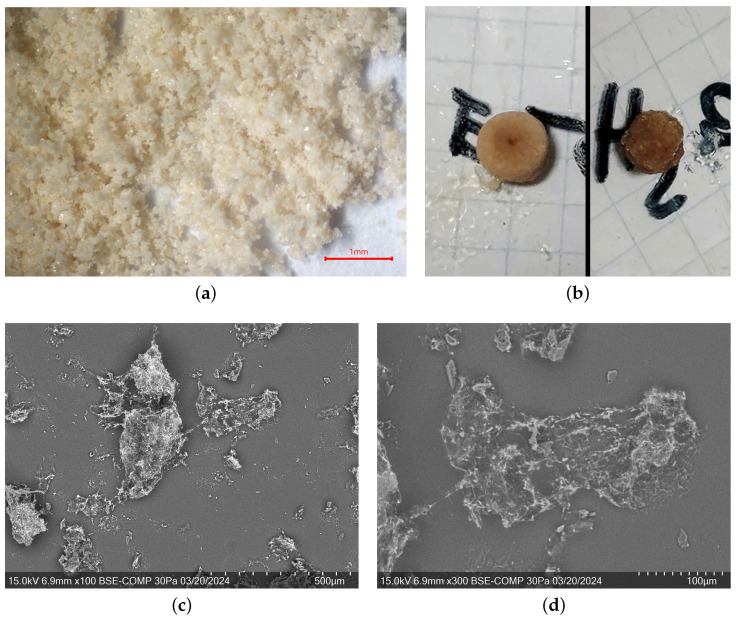

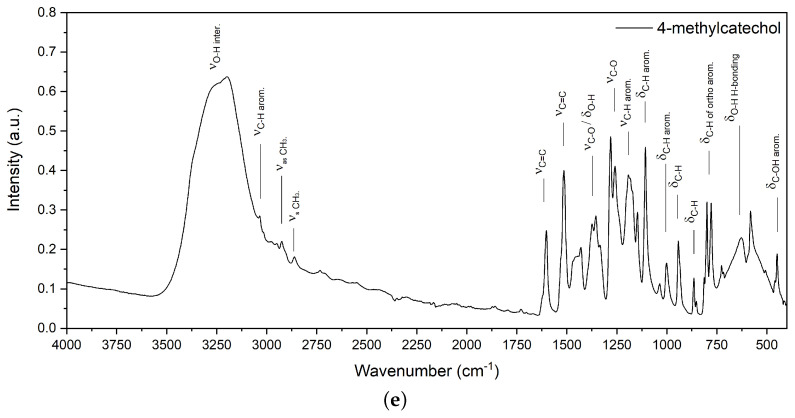
Optical (**a**,**b**) and scanning electron (**c**,**d**) microscopies of 4-methylcatechol re-crystallization attempts. On the subfigure (**e**) the FTIR spectrum of 4MEC is reported. Abbreviation: δ: bending vibration; $\nu_{s}$: symmetric stretching vibration; $\nu_{as}$: asymmetric stretching vibration.

4-methylcatechol (4MEC) directly sampled from the flask exhibiting a polycrystalline nature (Figure 1a,c,d), later confirmed by X-ray powder diffraction (XRPD). For this reason, XRPD data collected from this sample were directly exploited for structural resolution attempts. Additionally, utilizing pure 4MEC ensured the absence of any solvent molecules within its structure, eventually aiding in the identification of potential solvates coming from recrystallized samples. The SEM images are of low quality, as the sublimation of the sample is observed inside the chamber due to the applied vacuum and the excitation due to the electron beam. Despite these issues, it was possible to observe small heterogeneous crystals, with a maximum size of approximately 10 μm to 15 μm, clearly too small for single crystal diffraction analysis. Moreover, the quality of the crystals was also far from being optimal, as the crystalline habit is not well defined. The crystallization behavior and morphological characteristics of 4MEC were investigated through various crystallization methodologies employing solvents in which 4MEC is soluble [36] (water, ethanol and acetone) and their 1:1 mixtures. Two sets of solutions were prepared: one underwent slow evaporation over the weekend, while the other set was concentrated on a heater until complete evaporation occurred. Both methodologies resulted in the formation of polycrystalline aggregates, an example of which is present in Figure 1b, confirming the impossibility to obtain large single crystals. It is noteworthy that the growth of polycrystalline agglomerates has been often observed during evaporation (Figure 1b), with heights reaching up to half a centimeter. In some cases, the surprising ability of building a symmetric single form comprising all the crystallizing material was often observed. The “donut” and the “meatball” in Figure 1b are two of the more strange and peculiar aggregate growths. This kind of supra-crystalline hierarchical aggregate is reported in scientific literature typically for inorganic aggregates [37,38,39]. In conclusion, no crystallization attempt has provided qualitatively better products than the material taken directly from the container. Prior to conducting the diffraction measurement, a FTIR spectrum (Figure 1e) of the material was measured to confirm its purity, absence of solvents, and to unveil the interaction of hydroxyl groups with the molecular neighborhood. At wavenumbers greater than 3400 cm^−1^, typical bands of free alcoholic functions (From 3650 cm^−1^ to 3600 cm^−1^) and the upper part of the band belonging to crystallization water (3600 cm^−1^ to 3200 cm^−1^) are not visible. Instead, a broad band ranging from 3400 cm^−1^ to 3200 cm^−1^ is observed, associated with the prevalence of intermolecular hydrogen bonds. In the range from 3600 cm^−1^ to 3400 cm^−1^, the band associated with intramolecular hydrogen bonds of neighboring OH groups is not observed, suggesting the presence of a network of exclusively intermolecular hydrogen bonds.

### 2.2. Crystal Structure of 4-Methylcatechol

**Figure 2:**
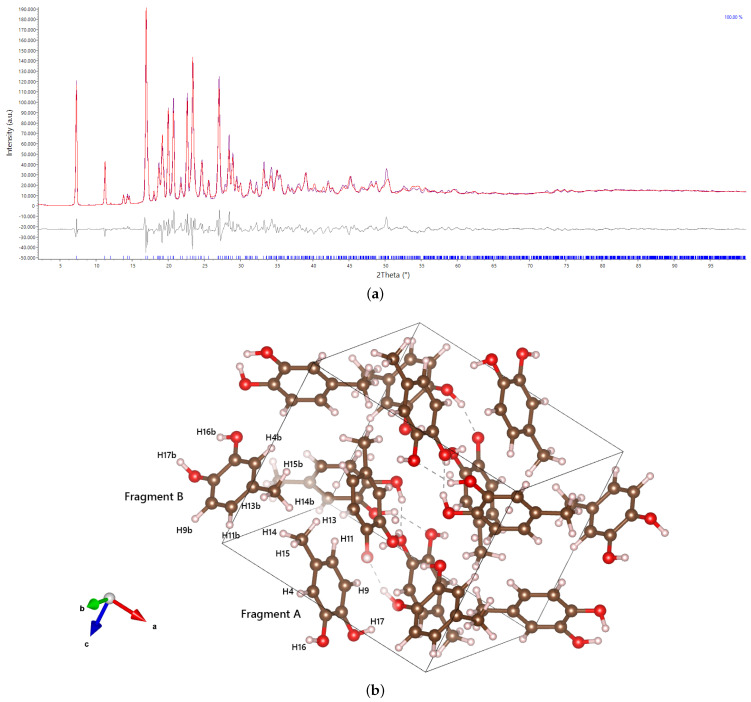
(**a**) Rietveld structural refinement (red) of 4-methylcatechol experimental XRPD data (blue); (**b**) Packing of 4-methylcatechol (C: brown; O: red; H: white).

XRPD data were collected in parafocusing geometry, revealing a well-defined powder pattern for 4MEC (Figure 2a), albeit with some evidence of preferred orientation corroborated by refinement data. Consistently, all recrystallized samples exhibited very similar diffraction patterns, confirming the absence of solvate formation and, at the same time, suggesting a lack of polymorphism under standard temperature and pressure conditions. The crystal unit cell dimensions for 4MEC, as determined by the Rietveld refinement in Figure 2a, and reported in more detail in Table 1, are as follows: a = 12.1689(14) Å, b = 10.3322(12) Å, c = 10.5141(12) Å, with angles α = 90°, β = 93.449(6)°, and γ = 90°. The volume of the unit cell is 1319.6(3) ${Å}^{3}$, with the $P2_{1}/c$ space group. Comparing the cell with those reported in the literature, a similarity can be observed with that of the catechol (Brown [40], resubmitted by Wunderlich and Mootz [41]), which has the same space group and has the b side approximately halved, as there is only one molecule in the asymmetric unit (CCDC codes: 1120673 and 1120674). Two molecules, named fragment A and B in Figure 2b are located in the asymmetric unit. Observing the structure along the c-axis, it is possible to recognize alternating hydrophilic and hydrophobic layers. The hydrophobic layers are composed of aromatic rings from fragment A, which arrange themselves in a staggered parallel manner with a distance between the centroids of 5.32 Å and an angle between the plane of one of the two parallel rings and the line identified by the centroids of the two rings of approximately 44°. These values suggest a far from frontal π–π stacking with a very weak interaction, as these parameters have optimal values of 3.8 Å and 20°, respectively [42]. Conversely, molecule B shows a T-shaped interaction together with another B fragment replicated by symmetry. A and B molecules interact each other only through hydrogen bonds on the hydrophilic side and the clustering of methyl groups on the other side, causing the presence of short distances due to steric hindrance. These weak contacts will be further commented in the Hirshfeld surface analysis section. During the structure determination process, all C−H hydrogen atoms were positioned according to the expected distances dictated by crystallochemistry, as bond angles are strictly governed by the hybridization of the corresponding carbon atoms. However, the positioning of hydrogen atoms associated with alcoholic groups, which is directed by the torsional angle around the C−O bond axis, proved to be unfeasible through Rietveld refinement due to the limited resolution of the technique [43,44]. To address this limitation, first principle calculations were conducted using Gaussian16 to optimize the positions of the hydrogen atoms and achieve a more accurate characterization of the hydrogen bonding network.

### 2.3. Hydroxyls Geometric Optimization and Hydrogen Bond Framework Analysis

**Figure 3:**
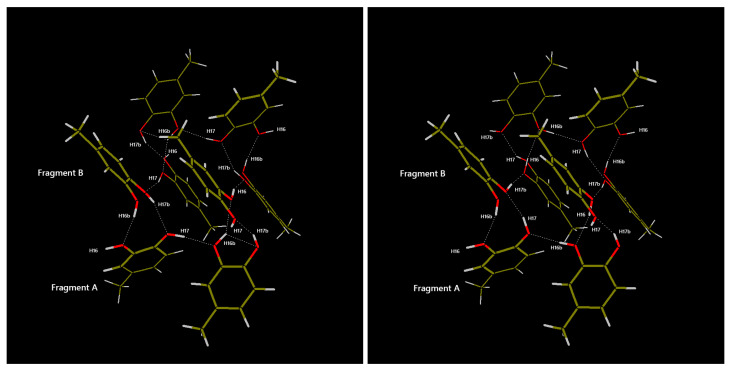
B3LYP/6-31G(d,p) optimized clusters of 8-molecule clusters of 4MEC: on the left the most stable Box 1, and on the right the alternative conformation Box 2 (C: brown; O: red; H: white).

To obtain a clear geometric and energetic view of the H-bond network, a geometric optimization of the hydroxyl hydrogen atoms was carried out on an eight-molecule cluster, suitably extracted from the 4MEC unit cell to completely represent all the possible H-bonds formed in the periodic structure. Several input files were generated, positioning the sixteen H hydroxyl atoms in the cluster using random values for each H−O−C−C torsion angle. The geometries of all the clusters were optimized, fixing all the O and C atoms (well determined experimentally by the XRPD data) and leaving all the hydrogen free to relax to minimize the cluster energy. Therefore, only the geometric parameters involving hydrogen atoms are commented. At first, the Hartree–Fock approach and the 3-21G basis set were exploited to identify the stable H-bond networks. All the generated models converged to the same two cluster conformations, named Box1 and Box2, with two possible H-bond conformations and different H-bond networks. These two stable clusters were further optimized by DFT calculations to obtain better H positioning and improved relative energy values. In Figure 3 both boxes are reported after the B3LYP/6-31G(d,p) optimization. In the figure on the left, Box 1 presents hydroxyl hydrogen atoms positioned to form hydrogen bonds in the central cluster that appear to rotate counterclockwise in the chosen viewpoint. Box 2, on the other hand, has an H-bond system that rotates clockwise. As can be seen in Figure 3 Box 1 and Box 2 have, respectively, 14 and 12 H-bonds per unit cell, suggesting marked energy differences. Their relative stabilities, at all tested levels of theory, are reported in Table 2 and can be used to choose the best hydroxyl groups conformation for the final Rietveld refinement.

Box 1 resulted as being more stable in the range from 34.4 kJ mol^−1^ to 45.7 kJ mol^−1^ than the one calculated for Box 2. Interestingly, the gap increases together with the level of calculation, as confirmation of the reliability of the approach. This difference is not negligible at room temperature and the H-bond network of Box 1 was thus used for the positioning of hydrogen atoms in the final Rietveld refinement, whose result is reported in Figure 3. It can be seen how the structure is, as expected, strongly dependent on hydrogen bonds, forming rather peculiar octagonal structures that involve all eight molecules in the unit cell. Each molecule of 4MEC participates in two octagonal systems, which results in an alternating layered arrangement visible looking at the structure along the c-axis. The formation of these hydrogen bonding systems causes the two molecules in the asymmetric unit to be very close to each other, which results in the formation of short contacts (i.e., H13···H14 = 1.70 Å, H14···H15 = 1.75 Å, H16···H17 = 1.62 Å and H14···H14b = 1.99 Å as defined in Figure 2b) between aliphatic and aromatic hydrogen atoms when they are replicated by symmetry. Comparing the structure of 4MEC with the one belonging to catechol, already mentioned in the previous section, a similar network of hydrogen bonds can be observed. However, having only one molecule in the asymmetric unit, there is a formation of distinct layers of hydrogen bonds, but not the formation of a three-dimensional network as instead happens in the case of 4MEC.

### 2.4. Hirshfeld Surface Analysis: Differences between the Two Molecules in the Asymmetric Unit

**Figure 4:**
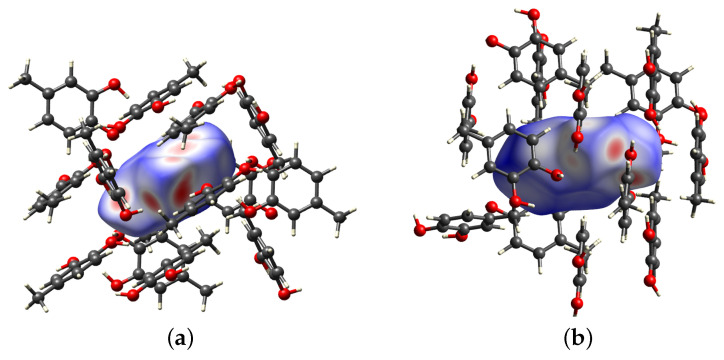
Hirshfeld surface analysis for fragment A (**a**) and fragment B (**b**) of the asymmetric unit (C: grey; O: red; H: white).

Both 4MEC fragments present in the asymmetric unit were subjected to Hirshfeld surface analysis in order to highlight any differences between the short contacts to which the two fragments are subjected, in particular how the two fragments contribute to the construction of the hydrogen bond network. Figure 4 shows the surfaces in heat maps for the two fragments: fragment A on the left and fragment B on the right. The scale of the heat maps ranges from blue, which represents regions in which the surrounding atoms are too distant to participate in intermolecular interactions, to red, where there are instead contacts between neighboring molecules.

It is evident that the two surfaces exhibit distinct characteristics, thus confirming marked differences in their contribution to the hydrogen bond network. Notably, fragment A demonstrates shorter contact distances, particularly within the region housing the diol system. In Figure 4b, fragment B, despite being a participant in the hydrogen network, has shorter interaction distances in the same area of the surface, characterized by smaller red spots.

**Figure 5:**
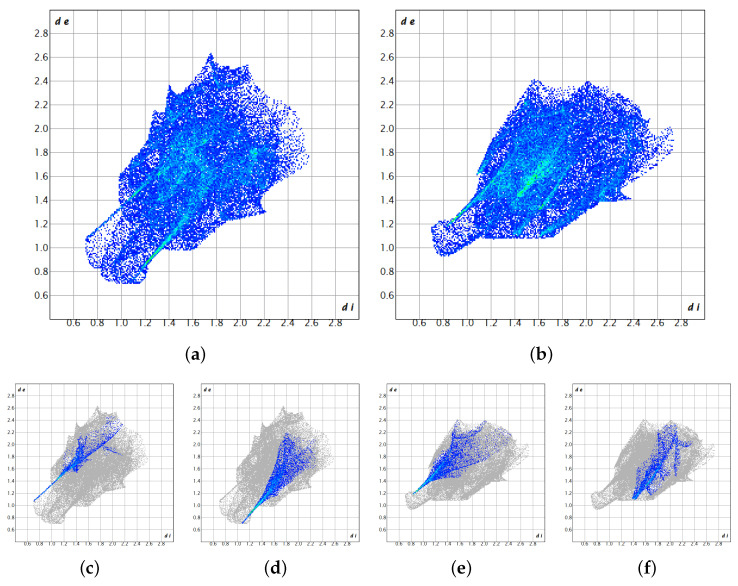
Fingerprint plot belonging to fragment A (left block) and fragment B (right block). On the top line (**a**,**b**), the two complete fingerprint plots are reported. On the bottom line, partial fingerprint plots are reported for H-O contacts (**c**,**e**) and O-H contacts (**d**,**f**).

Figure 5 presents the fingerprint plots computed for fragments A and B, depicted in the left and right columns, respectively. The plots offer visual confirmation of observations made regarding the Hirshfeld surface, indicating the distinct roles played by the fragments in forming the hydrogen bond network. This discrepancy becomes notably apparent upon examination of the specific H···O and O···H interactions within each fragment. For fragment A, two clearly delineated spikes indicate short contacts, whereas the distances for fragment B are comparatively longer. The fingerprint plots filtered by element for fragments A and B confirm an inefficient π–π stacking (C−C interactions 0.0%), which is the reason why they have been omitted in Figure 5. Meanwhile, the H−H interactions are clearly visible and dispersed like a homogeneous cloud at the center of the plot, confirming the existence of the hydrophobic clustering, especially of methyl groups. The choice of the selected cell and space group is then further supported by the different role played by the two molecules in the asymmetric unit in the construction of the crystalline network. Finally, it explains why every attempt at refining the 4MEC X-ray powder diffraction data starting from the crystal structure of catechol has failed. Similarly, the coexistence of two stable conformations for the H-bond network explains why large single crystals are not created, since at room temperature both conformations can be populated in solution, driving the crystal growth toward the construction of supra-crystalline hierarchical aggregates (Figure 1b).

## 3. Materials and Methods

### 3.1. Materials and Sample Preparation

All of the chemicals were purchased from Merck (Merck KGaA, Darmstadt, Germany). Efforts towards crystallization from solution were undertaken exploiting various solvents, namely water, ethanol, and acetone and their 1:1 mixtures. In each instance, a 50 mg quantity of 4-methylcatechol was dissolved in 2 mL of the respective solvent. Following thorough stirring to ensure complete dissolution, the solutions were allowed to settle in watch glasses and beakers. Notably, crystallization attempts did not yield single crystals in any case. It is noteworthy that upon complete solvent evaporation, polycrystalline agglomerates were observed in the case of pure solvents, while amorphous glasses were obtained by the evaporation of the 1:1 mixture solutions. Subsequently, both the original powder sampled from the bottle and the resultant agglomerates from pure solvent evaporation underwent gentle grinding before being subjected to measurement via X-ray powder diffraction. The best powder pattern resulted the “as purchased” sample and that one was used for structure solution and analysis.

### 3.2. Structure Solution by X-ray Powder Diffraction

The 4-methylcatechol samples underwent characterization using a Bruker D8 Advance diffractometer equipped with a copper $K_{\alpha }$ X-ray source and a LynxEye XE-T detector. The source operated at 40 mA current and 40 kV electric potential, with a goniometer radius set to 280 mm under standard measurement conditions. Soller slits with a 2.5° angle were used on both primary and secondary optics to mitigate the impact of axial divergence. For indexing, structure solution, and final Rietveld refinement, data collection was performed in Bragg–Brentano geometry. Samples were gently ground and positioned in standard polycarbonate sample holders. Variable width slits were employed as primary optics to maintain a constant 17 mm wide irradiated sample portion. Patterns were collected within an angular range from 2° to 100° in 2θ with a step-size of 0.02°/step and a collection time of 4 s/step. Indexing was carried out employing EXPO2014: cell parameters were determined using the N-TREOR09 and DICVOL06 algorithms, and the space group was selected based on agreement factors and the absence of non-indexed reflections. Structure solution was performed concurrently on Topas and EXPO using the simulated annealing method in direct space. Hydroxyl H atoms were placed according to the more stable position identified by first principle calculations. Crystallographic data have been submitted to CCDC with deposition number 2342610. Structure visualization and void analysis were performed exploiting VESTA 3.5.8 software [45] and Moldraw [46].

### 3.3. First Principles Calculations

The geometric optimization of the hydroxyl hydrogen atoms was carried out with the Gaussian16 software [47], using the Hartree–Fock method with the 3-21G basis set for approximate positioning. Starting from the optimized structure obtained, DFT calculations were carried out with B3LYP and 6-31G(d,p) basis set level of theory for fine positioning [43,48]. Using the latter setup, single-point energies of the different configurations of the hydrogen bond framework were compared to select the most stable one. To further support the obtained results, the relative stability and geometric optimization were investigated at the B3LYP-GD3/def2PVTZ level. Moreover, the Zero-Point Energy (ZPE) correction was calculated on the B3LYP/6-31G(d,p) geometry. The clusters and their chemical/crystallographic description are described in the result section.

### 3.4. Hirshfeld Surface and the Energy Frameworks Analyses

The Hirshfeld surfaces, fingerprint plots, and energy frameworks were computed through first principle calculations using CrystalExplorer 17.5 [49]. High-resolution settings were applied for the calculation of Hirshfeld surfaces in both structures. Wavefunctions and pairwise interactions for energy framework estimation were determined for each molecule using Gaussian16 [50] with the B3LYP density functional theory (DFT) method and the 6-31G(d,p) basis set, being the standard setup of CrystalExplorer. The tube size scale for energy framework illustrations was set to 80, with an energy cut-off value of 0 kJ mol^−1^. Interaction energies between each molecule and its chemical neighborhood were computed. The sum of lattice energy for each individual molecule was derived as one-half the product of the number of symmetry equivalent molecules in the cluster and the total energy, as outlined in Thomas et al. [51].

### 3.5. Infrared Analysis

FTIR analysis was carried out using a Nicolet iN10 instrument by Thermo Fischer Scientific, employing the attenuated total reflectance (ATR) module. Prior to each measurement, spectra of the air background were recorded. Spectra were acquired with a resolution of 4 cm^−1^ over a wavenumber range from 4000 cm^−1^ to 400 cm^−1^, with a total of 32 scans. The interpretation of the spectra and band assignment were carried out referring to G. Socrates [52].

### 3.6. Scanning Electron Microscopy and Optical Microscopy

Observations via electron microscopy and energy-dispersive X-ray spectroscopy (EDS) were performed using a Hitachi FLEXSEM 1000 microscope. Electron imaging utilized a tungsten filament at 15 kV without coating the 4-methylcatechol powders. Optical images with high resolution were obtained using a STEMI 508 microscope equipped with 1× frontal optics, a VisiLED ring light, and a 20 MPx SONY sensor camera.

## 4. Conclusions

The structure of 4-methylcatechol was solved combining X-ray powder diffraction and first principle calculations, since suitable single crystals were not obtained despite many crystallization attempts. The approach used in this paper paves the way for solving the crystal structures of compounds for which access to a single crystal is not feasible. Furthermore, the positioning of hydrogen atoms in hydroxyl groups exploiting first principle calculations constitutes an overcoming of the experimental limit related to the inability to observe the exact positions of hydrogen atoms through X-ray diffraction without resorting to neutron diffraction. Two molecules, with markedly different H-bond patterns, are present in the asymmetric unit, confirming that simpler and more frequently occurring crystal packing motifs with a single molecule in the asymmetric unit are not stable. A peculiar hydrogen bond network with hydroxyl nests formed by adjacent octagonal framework of hydrogen bond resulted the driving force of the crystal growth. Interestingly, the short contacts suggest strong intermolecular interactions, further confirmed by strong inter-crystalline aggregates observed in the microscopic optical images, indicating the growth, in many crystallization attempts of single aggregates taller than half a centimeter and with peculiar shapes. Because of their stability, the presence of H-bond nests can be inferred in solution, especially in a hydrophobic environment.

Such features explained why the crystal structure was, up to date, never solved, despite the very simple chemical formula C_7_O_2_H_8_ of 4MEC. By observing the complex network of hydrogen bonds, future applications can be envisaged, coupling 4MEC with active pharmaceutical ingredients (APIs) to form cocrystals containing an antioxidant to reduce side effects of the drug. Once more, solid-state chemistry teaches us how a single methyl group in 4MEC causes such dramatic difference with catechol. In fact, differently from 4MEC, catechol was solved in 1966 as a single crystal and shows one molecule in the asymmetric unit and a standard layered structure with alternating hydrophobic and hydrophilic zones.

## Figures and Tables

**Table 1:** Crystal data and structure refinement parameters for 4-methylcatechol.

Chemical formula	C_7_H_8_O_2_	Formula weight	124.13 g mol^−1^
Crystal system	monoclinic	Space Group	P $2_{1}$/c
a	12.1689(14) Å	α	90°
b	10.3322(12) Å	β	93.449(6)°
c	10.5141(12) Å	γ	90°
Volume	1319.6(3) ${Å}^{3}$	Z	8
T	293.15 K	$\lambda_{\mathrm{Cu}\;K_{\alpha }}$	1.54175 Å
$\rho_{calc}$	1.250 g cm^−3^	μ	0.753 mm^−1^
$F_{000}$	528.0	$F_{000^{\prime }}$	529.74
$R_{p}$	7.7857	$R_{wp}$	10.9931
CCDC number	2342610		

**Table 2:** First principles energies relative to Box1 H-bond network.

Level of Theory	Box1 Energy (kJ mol^−1^)	Box2 Energy (kJ mol^−1^)
HF/3-21G	0.0	+34.4
B3LYP/6-31G(d,p)//HF/3-21G	0.0	+39.9
B3LYP/6-31G(d,p)	0.0	+43.7
B3LYP-GD3/def2PVTZ	0.0	+45.7
ZPE-corrected B3LYP/6-31G(d,p)	0.0	+43.2

ZPE: Zero-Point Energy.

## Data Availability

Crystallographic information of the title compound were deposited in the CCDC’s database. The associated Refcode is 2342610.
